# Nutritional Deficiencies in Celiac Disease: Current Perspectives

**DOI:** 10.3390/nu13124476

**Published:** 2021-12-15

**Authors:** Anil K. Verma

**Affiliations:** Celiac Disease Research Laboratory, Department of Pediatrics, Università Politecnica delle Marche, 60123 Ancona, Italy; a.k.verma@pm.univpm.it

Gluten-induced T-cell-mediated immune response damages the villous structure that significantly affects the functioning of the small intestinal mucosa. Due to continuous mucosal damage, the villous cannot sufficiently absorb the nutrients from the food, ultimately leading to severe nutritional deficiencies in patients with celiac disease (CD) [1]. A complete gluten-free diet (GFD), the only accepted treatment of CD, gradually neutralizes the immune response and regenerates the damaged villi [2]. In comparison with gluten-containing food that is a major source of vitamins, iodine, dietary fiber, proteins, and minerals, the gluten-free alternative grains (rice, maize, etc.) and processed gluten-free food are inadequately nutritious and cannot compensate the necessary nutritional requirements [3,4]. Hence, strict adherence to GFD causes a continuous nutritional imbalance in CD patients, abolishes their quality of life (QOL), and eventually leads to moderate to severe disorders such as iron deficiency anemia and depression [5].

This Special Issue was planned to gather information about new prospects of nutritional deficiencies in CD. Articles related to the nutritional deficiencies in CD were invited. In total, 12 articles (8 original and 4 review articles) were published.

A substantial number of studies already correlated the consumption of Ultraprocessed food (UPF) with different diseases. However, very few studies assessed the side effects of UPF on oxidative/antioxidants balance. In the first original article, Nestares et al. evaluated the influence of UPF consumption on oxidative stress, antioxidant capacity, and inflammatory signaling in celiac children consuming a GFD. The authors reported that CD children who consume more UPF and perform less physical activity display higher levels of oxidative stress and some pro-inflammatory cytokines, regardless of the duration of GFD [6].

A considerable number of CD patients are often diagnosed with autoimmune and non-autoimmune thyroid disorders [7,8]. Iodine is an essential micronutrient for the synthesis of thyroid hormones, and its absorption occurs in the small intestine. Iodine malabsorption contributes to non-autoimmune thyroid disorders in CD. However, this hypothesis has never been investigated. In the second original study, probably the first study in the CD literature, Delvecchio et al. investigated the iodine excretion after proper dietary recommendations. The authors reported that the school-age CD patients have iodine deficiency during the diagnosis that partially recovers after one year of GFD [9].

A strict GFD limits the food choices for CD patients influencing them to choose foods with a high caloric content and a higher proportion of fat and protein. It is well known that commercial gluten-free products often have compromised nutritional quality, compared with their gluten-containing equivalents [3,4]. In the third original contribution, a cross-sectional age- and gender-matched study, Ballestero-Fernández et al., probably in a first study of its kind, investigated the nutritional status of the Spanish CD patients. The authors had a complete evaluation of the nutritional status of the Spanish adults diagnosed with CD and found an equivalent nutritional status in celiac adults, as compared with healthy volunteers, with the dietary deviations found similar to those of the Spanish population [10].

The severe acute respiratory syndrome coronavirus 2 (SARS-CoV-2)-related coronavirus disease of 2019 (COVID-19) pandemic has terribly been plaguing humankind since December 2019 [11]. CD has also not been untouched from this extremely challenging situation [12]. Regardless, a strict GFD reduces the QOL of CD patients [5]. However, following a GFD may have been more difficult during this pandemic. In an important original study (online survey), using an online ad hoc and validated questionnaire, Bascuñán et al. investigated perceptions about the general pandemic effects, current clinical conditions and dietary characterization, adherence to GFD, and mental health (anxiety and depression) in patients with gluten-related disorders including CD. The authors reported a higher level of depression and anxiety in celiac patients compared with the general population. However, this study does not clarify if the observed differences were due to the presence of disease or the pandemic [13].

In another timely needed original study, Falcomer et al. evaluated the QoL of Brazilian celiac patients during the course of the COVID-19 pandemic. The authors concluded that although the COVID-19 pandemic has historically posed a challenge for the Brazilian population, this period was not associated with a negative impact on Brazilian CD individuals’ QoL [14].

The most frequent extra-intestinal manifestation of CD is iron deficiency anemia (IDA). The iron absorption occurs primarily in the proximal duodenum section that is typically destroyed in CD. This destruction reduces iron absorption [15], although oral options such as products containing ferrous sulfate (FS) as an iron replacement are available. Poor tolerability of such oral supplements is frequent in patients with CD. In a review article based on iron deficiency anemia in CD, Talarico et al. comprehensively discussed oral ferrous supplements options and concluded that iron-based products (bivalent or trivalent) are well tolerated, compared with FS, but they may be less effective in correcting IDA, especially with CD patients [16].

Autoimmune thyroid diseases (AITDs) such as Hashimoto’s thyroiditis (HT) and Graves’ disease (GD) are the major causes of hypo and hyperthyroidism and frequently coexist with CD [17]. In contrast to iron deficiency, AITDs worsen preexisting thyroid dysfunction due to the decreased activity of the heme-dependent thyroid peroxidase [18]. In the second review article, Starchl et al. rigorously reviewed the literature to establish knowledge of CD and its relation to AITDs, as well as the influence of iron, vitamin D, and the gut microbiota on their onset and progression. The authors found the existence of a significant thyroid–gut axis, indicating effects of the gut microbiome on the immune system and the absorption of micronutrients, as well as on thyroid function [19].

Replacement of wheat and other related grains with gluten-free equivalents is associated with the increased consumption of trans fats, salt, sucrose, and phosphorus. These unhealthy food options can lead to the development of metabolic disorders and other cardiovascular complications [20].

In another original article, Gładyś et al. assessed the nutritional value of a GFD in adult CD patients before and after one year of standard dietary education. The authors found that 38% of CD patients do not adhere to a standard GFD. The authors also reported that CD subjects consume more unhealthy food; therefore, the role of dietitians in the treatment of CD must be increased [21].

On the other hand, it is important for healthcare workers, researchers, and dieticians to have enough knowledge about CD. In recent years, insufficient knowledge of CD among healthcare workers has been reported [22,23]. However, only a limited number of studies have been reported thus far on this important issue. In an original study, using an online survey in Poland, Dembiński et al. assessed the knowledge of healthcare professionals concerning nutritional deficiencies in patients with CD. The authors found that healthcare professionals and medical students in Poland have insufficient knowledge of the risk of nutritional deficiencies in patients with CD [24].

Following a long-term strict GFD often causes nutritional deficiencies [25]. In the third review article, Cardo et al. gave an updated vision on nutritional deficiencies in adult celiac patients following a GFD. The authors concluded that it is vital to carry out a continuous and personalized follow-up of celiac patients from the moment of diagnosis. For this supervision, the role of celiac expert nutritionists is essential [26].

Although IDA usually reverts with a GFD, some patients show persistent IDA. Recent studies suggest an association between the rs855791 polymorphism in the TMPRSS6 gene and persistent IDA in adults with CD [27,28]. In an interesting original study, Urbaszek et al. assessed the potential link between rs855791 and persistent IDA in pediatric patients with CD. The authors, probably for the first time, reported the prevalence of rs855791 genotypes in children with CD. The study suggests that persistent IDA is uncommon in pediatric patients with CD [29].

Iron deficiency (ID) with or without anemia is a common complication in both children and adults with CD, causing significant morbidity and impairment of health-related quality of life (HRQL) [5]. In a fourth comprehensive review article, Montoro-Huguet et al. remarkably covered all the important aspects of ID and CD. The authors expansively discussed the prevalence of CD among patients with anemia and vice versa. This review article provides healthcare professionals an algorithm based on the severity of symptoms, its impact on the HRQL, the tolerance and efficiency of oral iron, and other factors that predict a poor response to oral iron, such as the severity of histological damage, poor adherence to GFD, and blood loss due to mucosal lesions [30].

Collectively, this Special Issue includes articles on various topics of nutrition and CD and provides updated knowledge and new prospects of nutritional deficiencies in celiac disease.
